# Malarial parasite diversity in chimpanzees: the value of comparative approaches to ascertain the evolution of *Plasmodium falciparum* antigens

**DOI:** 10.1186/1475-2875-12-328

**Published:** 2013-09-17

**Authors:** M Andreína Pacheco, Michael Cranfield, Kenneth Cameron, Ananias A Escalante

**Affiliations:** 1 Center for Evolutionary Medicine and Informatics, The Biodesign Institute, Arizona State University, Tempe, Arizona, USA; 2 Gorilla Doctors, Wildlife Health Department, University of California Davis, Davis, USA; 3 Wildlife Health Program, Wildlife Conservation Society, Brazzaville, Republic of Congo; 4 School of Life Sciences, Arizona State University, Tempe, Arizona, USA

**Keywords:** Antigen, Chimpanzee, Co-evolution, Circumsporozoite protein, Dihydrofolate reductase-thymidylate synthase, Malaria, Merozoite surface protein 2, Mitochondrion genome, *Pan troglodytes*, Parasite evolution, *Plasmodium* phylogeny, *Plasmodium*, *Plasmodium falciparum*, Var2CSA

## Abstract

**Background:**

*Plasmodium falciparum* shares its most recent common ancestor with parasites found in African apes; these species constitute the so-called Laverania clade. In this investigation, the evolutionary history of *Plasmodium* lineages found in chimpanzees (*Pan troglodytes*) was explored.

**Methods:**

Here, the remainders of 74 blood samples collected as part of the chimpanzees’ routine health examinations were studied. For all positive samples with parasite lineages belonging to the Laverania clade, the complete mitochondrial genome (mtDNA), the gene encoding dihydrofolate reductase-thymidylate synthase (*dhfr-ts*), the chloroquine resistance transporter (*Pfcrt*), the circumsporozoite protein (*csp*), merozoite surface protein 2 (*msp2*), and the DBL-1 domain from *var2CSA* were amplified, cloned, and sequenced. Other *Plasmodium* species were included in the mtDNA, *dhfr-ts*, and *csp* analyses. Phylogenetic and evolutionary genetic analyses were performed, including molecular clock analyses on the mtDNA.

**Results/Conclusions:**

Nine chimpanzees were malaria positive (12.2%); four of those infections were identified as *P. falciparum*, two as a *Plasmodium reichenowi-*like parasite or *Plasmodium* sp., one as *Plasmodium gaboni*, and two as *Plasmodium malariae.* All *P. falciparum* isolates were resistant to chloroquine indicating that the chimpanzees acquired such infections from humans in recent times. Such findings, however, are not sufficient for implicating chimpanzees as an animal reservoir for *P. falciparum*.

Timing estimates support that the Laverania clade has co-existed with hominids for a long-period of time. The proposed species *P. gaboni, Plasmodium billbrayi*, and *Plasmodium billcollinsi* are monophyletic groups supporting that they are indeed different species.

An expanded CSP phylogeny is presented, including all the Laverania species and other malarial parasites. Contrasting with other *Plasmodium*, the Laverania *csp* exhibits great conservation at the central tandem repeat region. *Msp2* and *var*2CSA, however, show extended recent polymorphism in *P. falciparum* that likely originated after the *P. reichenowi-P. falciparum* split. The accumulation of such diversity may indicate adaptation to the human host. These examples support the notion that comparative approaches among *P. falciparum* and its related species will be of great value in understanding the evolution of proteins that are important in parasite invasion of the human red blood cell, as well as those involved in malaria pathogenesis.

## Background

Despite the extraordinary progress made in malaria control, there are still 200 million clinical cases per year worldwide, with more than 2.6 billion people living at risk of infection [1,2]. Part of the challenge emerges from the fact that the four *Plasmodium* species commonly found infecting humans exhibit noticeable differences in their clinical manifestations, geographic distributions, and basic biological characteristics [3]. Such differences are explained, at least in part, by their independent origins as human parasites [4,5]. Indeed, phylogenetic studies have shown that host switches have been common among primate malarial parasite lineages and that they were important in the origin of human malarias [6-13].

Without a doubt, primate malarial parasites have a broader host range than previously suspected. Evidence of host switches in modern times are found in the case of the macaque parasite *Plasmodium knowlesi* infecting humans in specific ecological settings [14,15] and the cases of *Plasmodium vivax-Plasmodium simium* and *Plasmodium malariae-Plasmodium brasilianum* that are shared between humans and several species of New World monkeys [12,16]. In addition, the human parasites *P. vivax* and *P. malariae* have been found in African apes [9,17]. It is worth noting that, whereas *P. knowlesi* is a zoonosis transmitted from macaques [15], there is no data of malaria transmission from non-human primates to humans in the other cases [18].

The research community had previously known only one species closely related to *Plasmodium falciparum*, *Plasmodium reichenowi* found in chimpanzees [5,19-22]. However, the discovery of new *Plasmodium* lineages in African apes that share recent common ancestors with *P. falciparum* and *P. reichenowi* unveiled a more complex evolutionary history [9-11,21-23]. Previous studies on these African ape malarias have extensively discussed the possibility of an animal reservoir for *P. falciparum* malaria [9,11,18,22]. Whereas this is an issue of considerable importance, it is not the only one. Indeed, the value of these non-human *falciparum*-like parasites for understanding the genetic diversity of *P. falciparum* has not received adequate attention [19,20,24]. In this investigation, data from new isolates of *Plasmodium* spp. found in chimpanzees (*Pan troglodytes*) are reported. This expanded dataset allowed exploring the problem of delimiting species in malarial parasites, as this issue relates to the origin of *P. falciparum*. Then, those lineages were used to investigate the evolution of three malarial antigens: 1) the gene encoding the circumsporozoite protein (CSP), a pre-erythrocytic malaria vaccine antigen; 2) the gene encoding MSP2, an erythrocytic stage malaria vaccine antigen; and 3) the *var2CSA* gene that has been associated with malaria in pregnancy. Overall, this study reports long-term functional constrains in some of these antigens (e.g. CSP), as well as patterns of high polymorphism within *P. falciparum* that have a relatively recent origin (*msp2* and DBL-1 domain from *var2CSA*), bringing insights on their importance in the human-*falciparum* relationship.

## Methods

### Samples and diagnostic

The chimpanzees are housed at the Jane Goodall Institute’s (JGI) Tchimpounga Chimpanzee Rehabilitation Center in the Republic of Congo (RC). Blood samples were collected throughout 2009-2010 by the veterinary staff as part of the chimpanzees’ routine health examinations following standards approved by the Pan Africa Sanctuary Alliance. The archived remainders of those blood draws were made available by JGI to Arizona State University (ASU) for malaria diagnostics. The samples were exported by the JGI under the RC CITES Export Permit No. 001 (Feb 2, 2010) and imported by ASU under U.S. CITES Import Permit No. 09US094332/9. Other samples used in this investigation were described elsewhere [9,13,25].

Genomic DNA was extracted from whole blood (approximately 200 μl) using the QIAamp® DNA Blood Mini kit (Qiagen, GmbH, Hilden, Germany) and each sample was screened for *Plasmodium* parasites by nested polymerase chain reaction (PCR), using primers for a 1200 bp fragment of the Cytochrome b (*cytb*) gene [13,25]. Details about the protocols are shown in the Additional files 1 and 2.

### Species diversity and phylogenetic analyses

Approximately 5,800 bp of the parasites’ mitochondrial genomes (mtDNA) and the gene encoding dihydrofolate reductase-thymidylate synthase (*dhfr-ts*) were amplified (Additional files 1 and 2). Independent alignments for nucleotide sequences of the mtDNA and *dhfr-ts* gene were made using ClustalX v2.0.12 and Muscle as implemented in SeaView v4.3.5 with manual editing. A list of the species included in phylogenetic analyses is provided in the Additional file 3; information about the species such as their basic biology, geographic distribution, and host-range can be found elsewhere [3]. Phylogenetic relationships were estimated using Maximum Likelihood (ML) methods as implemented in PhyML v3.0 [26] and Bayesian methods using MrBayes v3.1.2 [27].

In the case of the mtDNA, both phylogenetic methods used a general time reversible + gamma model (GTR + G) because it best fit the data as estimated by MEGA v5.0 [28]. The reliability of the nodes in the ML tree was assessed by the bootstrap method with 200 pseudo-replications. In the case of Bayesian analysis [27], each of the three mitochondrial genes plus the non-coding regions were used as separate partitions [25]. Bayesian support for the nodes was inferred in MrBayes using 8 ×10^6^ Markov Chain Monte Carlo (MCMC) steps, and after convergence was reached, the 50% of the sample as a burn-in was discarded. Sampling was performed every 100 generations. Convergence is reached after the average standard deviation of the posterior probability is below 0.01 and the value of the potential scale reduction factor (PSRF) is between 1.00 and 1.02 [27]. In this analysis, all the lineages reported elsewhere were also included (Additional file 3) [9,13,25]. As a comparison, the genetic divergences in the mtDNA among and within different *Plasmodium* species were estimated using the Kimura 2-parameter model as implemented in MEGA v5.

Similar procedures were followed when estimating the phylogeny of *dhfr-ts*. A difference was that Bayesian support for the nodes was inferred in MrBayes using 3 ×10^6^ Markov Chain Monte Carlo (MCMC) steps. In the *dhfr-ts* analysis, sequences from different *Plasmodium* species available in GenBank were also included (see Additional file 4).

### Estimation of divergence times

Time trees were estimated using BEAST v1.6 [29] on the nucleotide mtDNA sequences (Additional file 3) using four categories: each gene (*cox1, cox3, cytb*) plus the non-coding regions as a separate partition. Relaxed clock methods were applied with a GTR + G model of substitution with heterogeneity among sites and in the four partitions, optimizing the parameters specifically for each of the partitions. Uniform distributions were used as priors for the calibration intervals and the MCMC was carried out until convergence and good-mixing of the samples were reached [29]. Since time estimates are sensitive to the assumptions and methods utilized [13,25,30-35], the exploration of different scenarios is always recommended [25]. In this investigation three events were used as calibration points, one based on a biogeographical landmark and two from fossils. The scenarios explored, based on the calibration points described in Additional file 1, were: (1) a combination of the relaxed 6-14.2 Mya calibration for *Papio-Macaca* divergence with the minimum of 20 Mya for the origin of lemur parasites [30,31]; (2) a combination of the 6-14.2 Mya calibration with the two the minimums: 23.5 Mya for the human/Macaca split [35] and 20 Mya; and (3) the most conservative time period of 6-8 Mya narrowly defined around the fossils of the *Papio-Macaca* divergence [32,34] with a minimum of 20 Mya for the origin of the lemur lineage [25].

#### *Plasmodium falciparum* chloroquine resistance transporter

In order to ascertain whether the *P. falciparum* strains found in chimpanzees were acquired from humans, the chloroquine resistance transporter (*Pfcrt*) was sequenced so that mutations conferring resistance to the drug could be detected [36]. A fragment of approximately 250 bp was amplified by PCR. This fragment contained all the mutations that have been associated previously with chloroquine resistance (C72**S**, M74**I**, N75(**E/K**) and K76**T**) [36]. Details about the PCR protocol are provided in the Additional files 1 and 2.

### Malaria antigens

#### Circumsporozoite protein (CSP)

CSP is the predominant protein found on the surface of the sporozoite [37-39], the haploid stage that is inoculated by the mosquito vector into the vertebrate host. In this investigation, the orthologous genes encoding the CSP protein in malarial parasites from chimpanzees and other non-human primate malarias were amplified (Additional files 1 and 2) and compared against those sequences available in the GenBank (see Additional file 4) [13,16]. The alignment of *csp* sequences was done using only the N and C-terminal regions since those can be accurately aligned [16], and the phylogenetic relationships were estimated following the methodology described for mtDNA with a general time reversible + invariant model (GTR + I). Bayesian support for the nodes was inferred in MrBayes using 2 ×10^6^ Markov Chain Monte Carlo (MCMC) steps.

#### Merozoite surface protein 2

Merozoite surface protein 2 (MSP2) is a highly abundant GPI-anchored protein of *P. falciparum* that is found exclusively in the Laverania clade [9,40-42]*.* The *msp2* alleles found in the extant *P. falciparum* populations have been grouped into two groups; so-called allele families 3D7 and FC27. Previous studies have suggested that these allele families, like others such as *msp1*, are maintained by balancing selection driven by the host immune responses [42-44]. This investigation compared the available *msp2* alleles in the GenBank (Additional file 4) from the two families against orthologs found in malarial parasites from chimpanzees (see Additional files 1 and 2). The alignment and phylogenetic analyses followed the same methodology as above; however, the data fitted a Hasegawa-Kishino-Yano + gamma model (HKY + G) using MEGA v5.0 [28]. Bayesian support for the nodes was inferred in MrBayes using 10 × 10^6^ Markov Chain Monte Carlo (MCMC) steps.

#### Var2CSA protein

Pregnancy-associated malaria (PAM) is related to the expression of a *var* gene, known as *var*2CSA [45-48]. Unlike others members of the *var* gene family, it is found in all *P. falciparum* parasites isolates [49]. Here, approximately 1,500 bp (out of 10,000 bp) of the *var*2CSA gene containing the complete Duffy binding-like 1 (DBL1) were amplified from seven malaria chimpanzee isolates. The PCR protocol and primers are described in the Additional files 1 and 2. Alignment of DBL1 *var*2CSA nucleotide sequences, obtained from chimpanzees and those available in the GenBank (Additional file 4), was done using ClustalX v2.0.12 and Muscle as implemented in SeaView v4.3.5 with manual editing. The phylogenetic relationship among DBL1 *var*2CSA alleles was estimated as explained above with a general time reversible + gamma model (GTR + G). In this case, Bayesian support for the nodes was inferred using 20 ×10^6^ MCMC steps.

The genetic polymorphism within each species (*P. falciparum* and the non-*falciparum* chimpanzee lineages) was studied by using the statistic π, which is the average number of substitutions per site between any two sequences. In addition, in order to explore the putative effect of natural selection, the average numbers of synonymous substitutions per synonymous site (dS) and non-synonymous substitutions per nonsynonymous site (dN) between a pair of sequences were calculated using Nei and Gojobori’s method [50] with the Jukes and Cantor correction. The difference between dS and dN was estimated, and the standard deviation was calculated using 1,000 pseudo-replications for dS and dN, as well as a two tailed Z-test on the difference between dS and dN also with 1000 bootstrap pseudo-replications [51], as implemented in MEGA v5.0 [28]. The null hypothesis was that dS = dN; or selectively neutral.

In addition, the mean (relative) evolutionary rate for each amino acid residue on the same alignment of 918 bp (306 aa) was estimated as implemented in MEGA v5.0 [28,51]. These rates were scaled such that the average evolutionary rate across all sites is 1. This means that sites showing a rate < 1 are evolving slower than average and those with a rate > 1 are evolving faster. These relative rates were estimated separating *P. falciparum* from *Plasmodium* sp. using the Jones-Taylor-Thornton model with a discrete Gamma + G distribution (4 categories). The maximum likelihood estimate of the gamma shape parameter was 0.3637 and the maximum Log likelihood for this computation was−4,469.104.

### GeneBank accession numbers published in this study

The sequences reported in this study were deposited in GenBank under the following accession numbers: (1) from JX893150 to JX893155 and KF040083 for mtDNA; (2) from JX893156 to JX893168 for *dhfr-ts* genes; (3) from JX899656 to JX899667 for *csp* genes; (4) from JX899668 to JX899677 for the DBL1 *var*2CSA genes, and (5) from JX893169 to JX893171, and JX899678 for *msp2* (see Additional file 4).

## Results

### Species diversity and phylogenetic analyses

Seventy-four chimpanzee (47 males and 27 females) blood samples were screened for *Plasmodium* parasites. Blood smears were collected, however, none of the samples were positive by microscopy. Nine chimpanzees were found positive (12.2%) by nested PCR using cytochrome b (*cytb*); seven males (7/47 = 14.8%) and two female (2/27 = 7.41%). Four of the nine positive samples were identified as *P. falciparum* (isolates 11, 14, 22 and 30) using BLAST, one a mixed infection of *P. reichenowi* and a *Plasmodium reichenowi* like parasite that will be further referred as *Plamodium* sp. (isolate 20), one as *Plamodium* sp. (isolate 70), one as *P. gaboni* (isolate 39), and two as *P. malariae* (isolate 16 and 56). One of the chimpanzees infected with *Plamodium* sp. was co-infected with *P. malariae* (isolate 70). Seven parasites’ mitochondrial genomes were generated from these samples: four *P. falciparum*, one *P. reichenowi*, one *Plasmodium* sp*.* closely related to *P. reichenowi*, and one *Plamodium gaboni* (Additional file 3). These new sequences were analysed together with comparable data from African ape malarias that were previously reported [9,21]; those included *P. gaboni* and a so-called *Plasmodium* sp*.* parasite lineage, as well as *Plamodium billcollinsi* and *Plamodium billbrayi*. The clade that contains *P. falciparum* and *P. reichenowi* and their sister lineages (*P. gaboni*, *P. billbrayi, P. billcollinsi* and *Plasmodium* sp.) will be collectively referred to as Laverania, a sub-genus used in classical taxonomy for just the species *P. falciparum* and *P. reichenowi*[52].

Genetic divergences among the mitochondrial genome of the *Plasmodium* species found in chimpanzees are shown in Table 1. As expected, the average genetic distance between the *P. gaboni* isolate sequence reported here and the one previously reported (FJ895307) was just 0.003 ± 0.001 with a total of 18/5,800 differences between the two isolates. This falls within the level of polymorphism observed in recognizable species such as *P. vivax* and *P. falciparum*. However, the average genetic distance between *P. billbrayi* and *P. gaboni* (0.0181 ± 0.0015 for the complete mtDNA, see Table 1), was comparable to the divergence observed between *P. vivax* and *Plasmodium cynomolgi* (0.0118 ± 0.0016, Table 1) and the one found among rodent malarias [25,53]. Furthermore, the average divergence estimated between the so-called *Plasmodium* sp*.* and *P. reichenowi* lineages, as well as to *P. billcollinsi* (see Table 1), were also comparable to those between well-recognized *Plasmodium* species found in mammals (Table 1).

**Table 1:** **Genetic divergences among different *Plasmodium *species**

		**Genetic distance (d ± Std Err.)**				
**Species**	**n**	**COX1**	**COX3**	**CYTB**	**COX1 + CYTB**	**complete mtDNA**
*P. vivax*	110	0.001 ± 0.0005	0.001 ± 0.0005	0.000 ± 0.0001	0.001 ± 0.0003	0.001 ± 0.0001
*P. vivax-P. cynomolgi*	1 vs. 2	0.014 ± 0.0029	0.014 ± 0.0033	0.013 ± 0.0030	0.012 ± 0.0018	0.012 ± 0.0016
*P. knowlesi*	59	0.001 ± 0.0004	0.002 ± 0.0007	0.001 ± 0.0004	0.001 ± 0.0003	0.001 ± 0.0002
*P. ovale wallikeri-P. ovale curtisi*	2	0.011 ± 0.0030	0.025 ± 0.0056	0.013 ± 0.0031	0.012 ± 0.0022	0.012 ± 0.0014
*P. chabaudi chabaudi*	7	0.001 ± 0.0005	0.002 ± 0.0009	0.001 ± 0.0003	0.001 ± 0.0003	0.001 ± 0.0003
*P. chabaudi adami*	2	0.004 ± 0.0016	0.005 ± 0.0025	0.004 ± 0.0017	0.004 ± 0.0013	0.003 ± 0.0007
*P. ch. chabaudi-P. ch. adami*	7 vs. 2	0.005 ± 0.0017	0.009 ± 0.0028	0.006 ± 0.0019	0.005 ± 0.0013	0.005 ± 0.0007
*P. billbrayi-P. gaboni*	4 vs. 2	0.020 ± 0.0036	0.038 ± 0.0067	0.021 ± 0.0040	0.021 ± 0.0029	0.018 ± 0.0015
*P. billbrayi-P. billcollinsi*	4 vs. 1	0.103 ± 0.0088	0.167 ± 0.0150	0.086 ± 0.0096	0.095 ± 0.0063	0.077 ± 0.0039
*P. reichenowi-Plasmodium* sp.*	2 vs. 1	0.019 ± 0.0034	0.026 ± 0.0055	0.017 ± 0.0087	0.018 ± 0.0027	0.015 ± 0.0016
*P. reichenowi-P. billcollinsi*	2 vs. 1	0.076 ± 0.0076	0.130 ± 0.0158	0.039 ± 0.0063	0.060 ± 0.0048	0.051 ± 0.0028
*P. reichenowi-P. falciparum* (H)	2 vs.101	0.032 ± 0.0047	0.055 ± 0.0085	0.025 ± 0.0049	0.029 ± 0.0032	0.025 ± 0.0018
*P. reichenowi-P. falciparum* (Ch)	2 vs. 4	0.032 ± 0.0043	0.056 ± 0.0083	0.026 ± 0.0048	0.029 ± 0.0031	0.026 ± 0.0021
*Plasmodium* sp.*-*P. falciparum* (Ch)	1 vs. 4	0.036 ± 0.0052	0.060 ± 0.0091	0.034 ± 0.0056	0.035 ± 0.0034	0.030 ± 0.0022
*P. falciparum* (Ch)*-P. billcollinsi*	4 vs. 1	0.062 ± 0.0072	0.130 ± 0.0143	0.051 ± 0.0072	0.057 ± 0.0043	0.051 ± 0.0027
*P. falciparum* (Ch)-*P. falciparum* (H)	4 vs.101	0.000 ± 0.0000	0.001 ± 0.0007	0.001 ± 0.0005	0.001 ± 0.0002	0.002 ± 0.0004
*P. falciparum* (H)	101	0.000 ± 0.0001	0.001 ± 0.0004	0.001 ± 0.0003	0.000 ± 0.0001	0.000 ± 0.0001

*Sequence GeneBank accession number GQ355476.

(H) Human samples and (Ch) chimpanzee samples.

**Figure 1:**
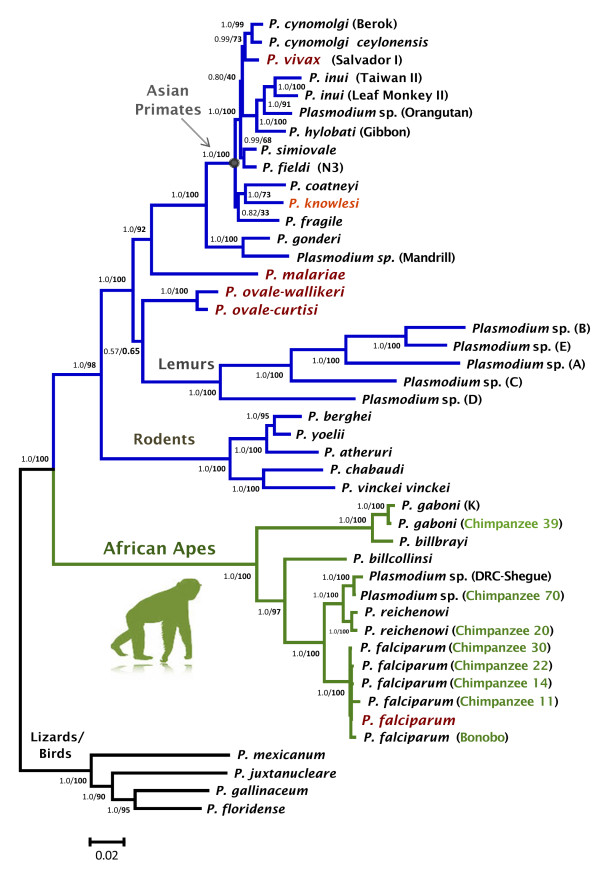
**Phylogenetic tree of *Plasmodium *spp. based on complete mitochondrial genomes.** Bayesian and Maximum Likelihood (ML) methods yielded identical topologies, only the Bayesian tree is shown. The values above branches are posterior probabilities together with bootstrap values (in bold) as a percentage obtained for the maximum likelihood tree (see methods). Human malaria parasites are labelled in red and the chimpanzee identification is labelled in green. Branches indicated in green correspond to the African apes malarias, branches in blue correspond to the other mammals *Plasmodium* parasites (rodents, lemurs, Asian primates, etc.), and black braches mean the out-group used in this study.

**Figure 2:**
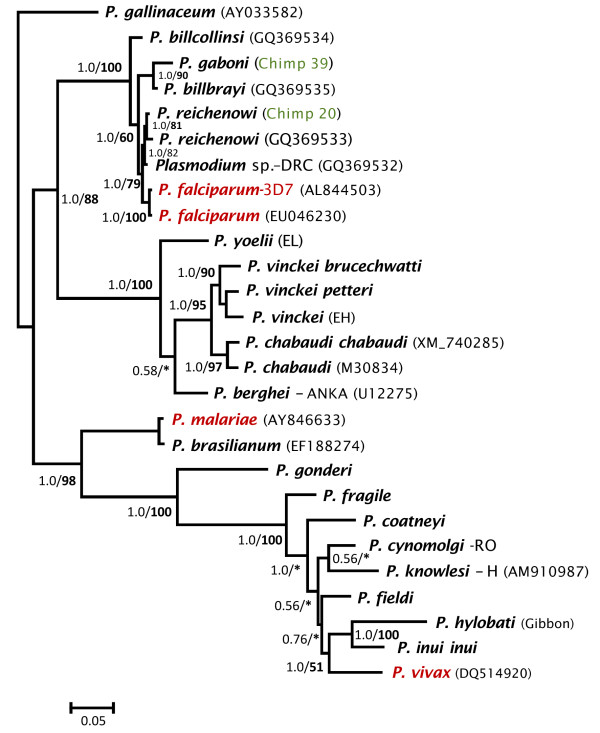
**Phylogenetic tree of *Plasmodium *spp. based on the gene encoding *dhfr-ts*.** Bayesian and Maximum Likelihood (ML) methods yielded identical topologies, only the Bayesian tree is shown. The values above branches are posterior probabilities together with bootstrap values (in bold) as a percentage obtained for the maximum likelihood tree (see methods). Human malaria parasites are labelled in red and the chimpanzee identification is labelled in green. * indicates a clade without support in the maximum likelihood tree.

Phylogenetic analyses were performed separately on the complete mtDNA (Figure 1) and *dhfr-ts* gene (Figure 2). Overall, both phylogenies were consistent, however, they differ in where the root is placed by using *Plasmodium gallinaceum* as an out-group. In the *dhfr-ts* phylogeny, the Laverania clade seems to share a recent common ancestor with rodent parasites. In both cases, maximum likelihood and Bayesian methods yield comparable results, indicating that the different Laverania lineages diverged following the same consistent pattern as one expected for different species (Figures 1 and 2). *Plasmodium gaboni* and *P. billbrayi* form a monophyletic group that share a common ancestor with the clade of *P. falciparum*-*P. reichenowi* (Figure 2)*.* Thus, both phylogenies reproduce almost identical evolutionary histories and all the Laverania lineages reported here. Furthermore, these lineages show divergences from their closest sister taxa that are comparable to those observed among well-established *Plasmodium* species. Overall, these results are consistent with previous studies that have identified multiple *Plasmodium* species in chimpanzees [9-11,21].

### Estimation of divergence times

**Figure 3:**
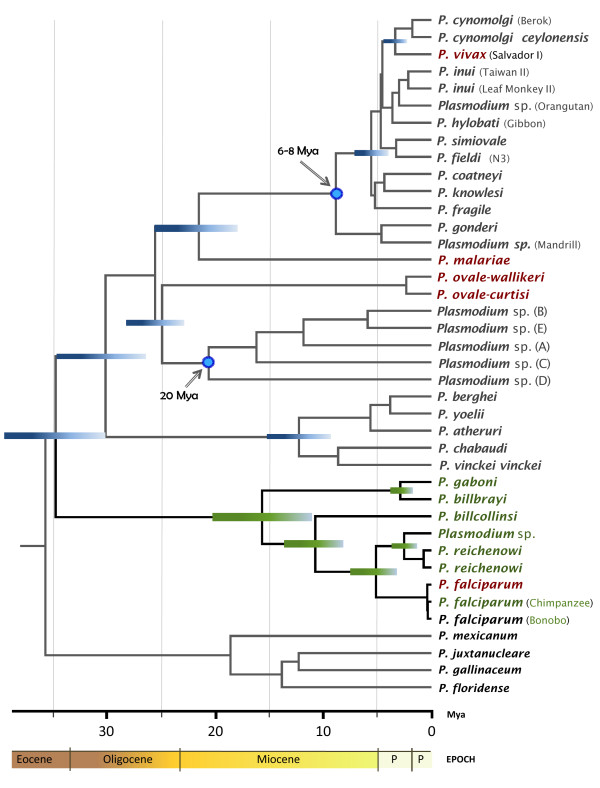
**Timetree of the divergence of malarial parasites.** Divergence times were estimated with BEAST using the most conservative scenario based on the minimum divergence of *Macaca* and *Papio* using fossils (6-8 Mya) and a minimum of 20 Mya for the origin of lemur parasites. Times are shown in millions of years ago (Mya).

The mtDNA phylogeny was used to estimate the time of origin of the African ape parasites (Figure 3). Table 2 shows the divergence times of *Plasmodium* splits in the Laverania clades as estimated by BEAST v1.6 [54] considering three different scenarios (see also Additional file 5 for the node numbers). As expected, the credibility intervals obtained using these scenarios overlap (Table 2). Under the first scenario, the estimates of the divergence times for the radiation of African ape malarias suggest that all five lineages might have originated between 13.09 and 22.93 million years ago (Mya); therefore, African ape parasites may have started to diverge long before the divergence of *Pan* and *Homo* (2.7-13.0 Mya) [55,56]. The *P. billbrayi-gaboni* split is estimated between 1.92-4.69 Mya and for *P. reichenowi*-*P. falciparum* between 4.02 and 7.84 Mya. Those estimates are older than those reported previously [9], but similar to those times estimated by Pacheco *et al*. [13,25], using fewer sequences but the same calibration points. However, regardless of the wide confidence interval, the time frames for the *reichenowi-falciparum* split are still consistent with the divergence of the genera *Pan* and *Homo*. Overall, these time estimates suggest that the Laverania species likely have been associated with hominids for a long time [9,13,25].

**Table 2:** **Divergence times of *Plasmodium *splits in the Laverania clade as estimated by BEAST**

**Calibrations (Mya)**		**Node 13: 6-14.2**		**Node 13: 6-14.2**		**Node 13: 6-8**	
				**Node 14: min = 23.5**			
		**Node 19: min 20**		**Node 19: min 20**		**Node 19: min 20**	
**Divergence**	**Node**	**Node Age (Mya)**	**95%CrI**	**Node Age (Mya)**	**95%CrI**	**Node Age (Mya)**	**95%CrI**
Radiation of African apes malaria	34	17.49	13.09-22.93	18.23	13.73-23.72	15.55	12.07-19.75
Split *P. billbrayi-P. gaboni*	27	3.16	1.92-4.69	3.31	2.03-4.87	2.8	1.73-4.06
Split *P. billcollinsi-*(*P. reichenowi-P. falciparum*)	33	11.98	8.73-16.17	12.48	8.97-16.63	10.63	7.85-13.79
Split *Plasmodium* sp.*-P. reichenowi*	31	2.75	1.80-3.97	2.84	1.84-4.08	2.41	1.53-3.41
Split *P. reichenowi*-*P. falciparum*	32	5.71	4.02-7.84	5.93	4.12-8.04	5.03	3.52-6.70
Radiation of Southerm Asia primates malaria	11	6.47	5.09-8.28	6.76	5.24-8.40	5.5	4.50-6.88
Radiation of rodents malaria	25	13.84	9.91-18.09	14.39	10.72-18.83	12.28	9.16-15.80
Radiation of *Plasmodium*in mammals	35	38.74	31.80-48.18	40.56	33.90-49.13	35.12	30.24-40.79

The node numbers are shown in the Additional file 5.

### *Plasmodium falciparum* chloroquine resistance transporter

The *P. falciparum* isolates found in the chimpanzees were acquired recently from humans. The mtDNA genome sequences clearly belong to *P. falciparum*[57] and all the isolates had *Pfcrt* mutations (allele CV**IET** from Africa/Southeast Asia) [36] associated with chloroquine resistance. It is also worth noting that all the other Laverania species lack those *Pfcrt* mutations associated with chloroquine resistance. This result was expected since those species are not exposed to this anti-malarial drug.

### Evolution of malaria antigens

#### Circumsporozoite protein

**Figure 4:**
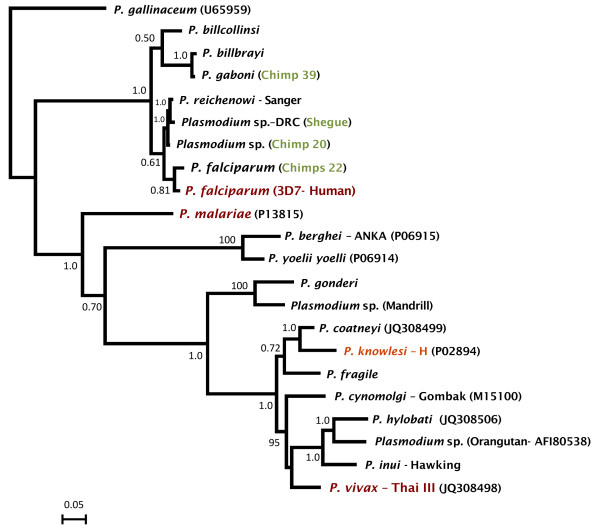
**Bayesian phylogenetic tree of chimpanzee *Plasmodium *based on the *csp *gene.** The values above branches are posterior probabilities (see methods). Human malaria parasites are labelled in red and the chimpanzee identification is labelled in green.

A phylogeny for the *csp* gene using the non-repetitive regions is depicted in Figure 4. It follows the trend evidenced by the mtDNA and *dhfr-ts* phylogenies in terms of showing that *P. falciparum* and all the *Plasmodium* sp. found in chimpanzees are part of a monophyletic group. However, there were differences in the relationship of the major clades when the *csp* gene tree was compared with the mtDNA and *dhfr-ts* phylogenies. Since only the non-repetitive region can be properly aligned [16], and not all phylogenetic analyses include the same number of species, those differences are likely the result of the limited phylogenetic information found in relatively short sequences.

**Figure 5:**
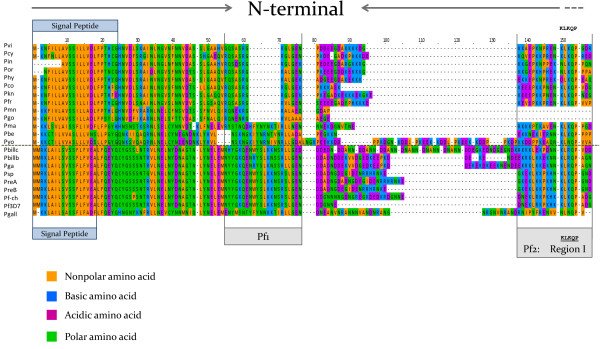
**Alignment of the N-terminal region for orthologous genes that encode CS protein in different *Plasmodium *spp.** Functional regions are described elsewhere [63,64]. The species names are abbreviated as follow: **Pvi**-*P. vivax* (Thai), **Pcy**-*P. cynomolgi* (Gombak), **Pin**-*P. inui* (Hawking), **Por**-*Plasmodium* sp. from orangutans (VS28), **Phy**-*P. hylobati*, **Pco**, *P. coatneyi*, **Pkn**-*P. knowlesi*, **Pfr**-*P. fragile*, **Pmn**-*Plasmodium* sp. from mandrill, **Pgo**-*P. gonderi*, **Pma**-*P. malariae*, **Pbe**-*P. berghei*, **Pyo**-*P. yoelli*, **Pbillc**-*P. billcollinsi*, **Pbillb**-*P. billbrayi*, **Pga**-*P. gaboni*, **Psp**-*Plasmodium* sp., **PreA**-*P. reichenowi* (Sanger), **PreB**-*P. reichenowi* from chimp 20, **Pf-ch**-*P. falciparum* from chimp 22, **Pf3D7**-*P. falciparum* strain 3D7, and **Pgall**-*P. gallinaceum*.

Usually, the central tandem repeat regions of the CS proteins exhibit extensive diversity in *Plasmodium* species [13,37-39]. Such diversity is evidenced in the number of repeats within *Plasmodium* species and diverging motifs, even among closely related species or within a single species [13,16,37-39,58-61]. However, unlike other malarial parasites [13,16,61], there is a strong conservation of the basic asparagine rich tandem motif among the species in the Laverania clade (Table 3). Specifically, the motifs PNAN and PNVD are present not only in *P. falciparum*[44,62], but also in all Laverania species. There are, however, a few differences. The motif PNVN is only found in *P. reichenowi* and *Plasmodium* sp. but not in *P. falciparum*. Likewise PNAD, a motif found in low number in *P. billcollinsi, P. billbrayi*, and *P. gaboni* is not present in *P. falciparum*[44,62]. Finally, two different asparagine rich motifs could be identified in *P. billcollinsi*; (PNANPN)_17_ and the less common (PNANPNPN)_2_. The frequency of the repeats appears in Table 3. Interestingly, *Plasmodium yoelii* and *P. billcollinsi* are the only species with repeats between the regions identified as **Pf1** and **Pf2** (Figure 5, Table 3) that can bind heparin/heparin sulphate binding sites (see below). In the case of *P. billcollinsi,* the repeat also involves an asparagine rich motif.

**Table 3:** CSP repetitive motifs and the polymorphism found in all Laverania species analyzed

**Species**	**NCBI No.**	**Repeat motifs**	**π**	**dS-dN (SE) / p (ZTest)**	**Repeat motif combination**
*P. billcollinsi*	JX899657	R1: [DDANN]_3_	0.040 (0.039)	−0.050 (0.049) 0.248 (1.161) dS = dN	***Pf1***-[1]_3_[2]_3_--***Pf2***--[3]_2_**4**[3]_4_**4**[3]_3_**4**[3]_5_5[3]_3_5-**PN**
		R2: [DNANN]_3_			
		R3: [PNANPN]_17_	0.078 (0.045)	0.470 ± 0.224 0.017 (−2.431) **dS** > dN	
		R4: [PNAD]_3_			
		R5: [PNANPNPN]_2_			
*P. billbrayi*	JX899659	R4: [PNAD]_4_	0.104 (0.053)	0.315 ± 0.203 0.023 (−2.298) **dS** > dN	***Pf2***---**4**[6]_2_**4**[6]_6_**4**[6]_6_**4**[6]_15_-**PN**
		R6: [PNAN]_29_			
*P. gaboni*	JX899661	R4: [PNAD]_2_	0.128 (0.058)	0.347 ± 0.246 0.021 (−2.338) **dS** > dN	***Pf2***---[6]_2_**7**[6]_2_**7**[6]_2_**4**[6]_7_**4**[6]_13_-**PN**
		R6: [PNAN]_26_			
		R7: [PNVD]_2_			
*P. reichenowi*		R6: [PNAN]_26_	0.164 (0.063)	0.211 ± 0.231 0.014 (−2.503) **dS >** dN	***Pf2***---[(6-7)]_4_6867[(6-8)]_3_[6]_17_-**PN**
		R7: [PNVD]_5_			
		R8: [PNVN]_4_			
*Plasmodium*sp. (isolate Shegue)	JX899658	R6: [PNAN]_27_			***Pf2***---[(6-7)]_4_6867[(6-8)]_5_[6]_16_-**PN**
		R7: [PNVD]_5_			
		R8: [PNVN]_6_			
*Plasmodium* sp. (isolate 20)	JX899660	R6: [PNAN]_26_			***Pf2***---[(7-6)]_4_7[6]_2_7[(6-8)]_2_[6]_18_-**PN**
		R7: [PNVD]_6_			
		R8: [PNVN]_2_			
*P. falciparum* (isolate 14)	JX899664	R6: [PNAN]_36_	0.131 (0.047)	0.224 ± 0.149 0.002 (−3.093) **dS** > dN	***Pf2***---[(6-7)]_3_[6]_11_7[6]_22_-**PN**
		R7: [PNVD]_4_			
*P. falciparum* (isolate 22)	JX899662	R6: [PNAN]_37_			***Pf2***---[(6-7)]_3_[6]_15_7[6]_19_-**PN**
		R7: [PNVD]_4_			
*P. falciparum* (isolate 30)	JX899663	R6: [PNAN]_37_			***Pf2***---[(6-7)]_3_[6]_15_7[6]_19_-**PN**
		R7: [PNVD]_4_			
*P. falciparum*(isolate 3D7)		R6: [PNAN]_40_			***Pf2***---[(6-7)]_3_[6]_19_7[6]_18_-**PN**
		R7: [PNVD]_4_			

**PN** are amino acids located at the end of the repetitive region. ***Pf1*** and ***Pf2*** are peptides described by Ancsin and Kisilevsky 2004 [63].

**Figure 6:**
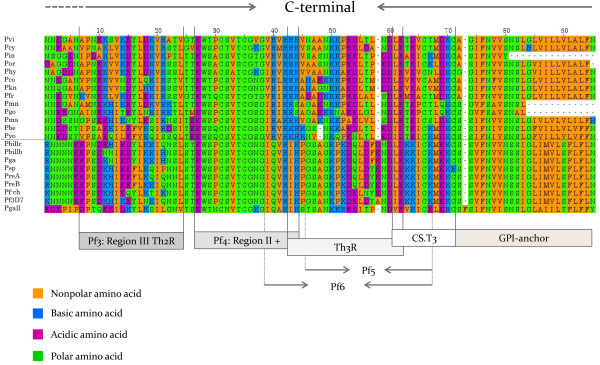
**Alignment of the C-terminal region for orthologous genes that encode CS protein in different *Plasmodium *spp.** Functional regions are described elsewhere [63,64]. The species names are abbreviated as follow: **Pvi**-*P. vivax* (Thai), **Pcy**-*P. cynomolgi* (Gombak), **Pin**-*P. inui* (Hawking), **Por**-*Plasmodium* sp. from orangutans (VS28), **Phy**-*P. hylobati*, **Pco**, *P. coatneyi*, **Pkn**-*P. knowlesi*, **Pfr**-*P. fragile*, **Pmn**-*Plasmodium* sp. from mandrill, **Pgo**-*P. gonderi*, **Pma**-*P. malariae*, **Pbe**-*P. berghei*, **Pyo**-*P. yoelli*, **Pbillc**-*P. billcollinsi*, **Pbillb**-*P. billbrayi*, **Pga**-*P. gaboni*, **Psp**-*Plasmodium* sp., **PreA**-*P. reichenowi* (Sanger), **PreB**-*P. reichenowi* from chimp 20, **Pf-ch**-*P. falciparum* from chimp 22, **Pf3D7**-*P. falciparum* strain 3D7, and **Pgall**-*P. gallinaceum*.

The alignment of the N and C-terminal domains of the *csp* gene is illustrated in Figures 5 and 6, respectively, incorporating information about functionally relevant parts of the protein [37,63,64]. The signal peptides (Figure 5) were predicted using the SignalP 4.0 Server [65]. The CS proteins from *P. billcollinsi*, *P. gaboni*, *P. billrayi*, and *P. reichenowi* show differences in a region of low complexity that is found prior to the so-called region I (see Figure 5). The region I (KLKQP) [64,66], contained in **Pf2**[63], is a protease cleavage site that plays a critical role in the processing of the CS protein in the mammalian host and exhibits high affinity to heparin sulphate found on the surface of liver cells or hepatocytes [37,38,63,64,66]. The motif KLKQP is conserved between *P. reichenowi* and *P. falciparum*, as is the case in many of the other *Plasmodium* species. However, three of the Laverania lineages differ, having the variant KL**R**QP instead of KL**K**QP, (specifically *P. billcollinsi* (Pbillc)*, P. billbrayi* (Pbillb)*,* and *P. gaboni* (Pgab)) (Figure 5)*.* Previously, the only known CSP sequences with variations in this region were the avian parasite *P. gallinaceum*, with the motif NLNQP, and the rodent parasite *P. yoelii,* with KLNQP [66,67]. Surprisingly, the two African Cercopithecidae parasites, *P. gonderi* (identified as Pgo in Figures 5 and 6) and the one from mandrills (Pmn) lack the region I (KLKQP). These results were reproduced not only by independent PCRs, but also by a perfect match in *Plasmodium gonderi* genomic data [68].

The other two regions in the CSP with capacity to bind heparin/heparin sulphate (HS) [63,64], in addition to **Pf2**-region I+, are a peptide in the N-terminal or **Pf1** and one in the C-terminal domain or **Pf4**-Region II+, see Figure 5 and 6[37,38,63,64,66]. **Pf1** is not considered critical [63] and is not conserved in *P. vivax* and related parasites [5,6], however, it is highly conserved among Laverania and the only avian CS protein sequence available [67] setting them apart from the other *Plasmodium* found in mammals (Figure 5). The **Pf4** with the so-called region II + [63,64] (Figure 6) is conserved among *Plasmodium* species and almost identical in the Laverania clade (EWSPCSVTCGNGIQVRIK) with few synonymous substitutions (dS = 0.1504 vs. dN = 0.0). The **CS.T3**, a T-cell epitope responsible for a CD4+ T-cell response that correlates with protection [64], is also identical among *P. falciparum* and the *Plasmodium* species from chimpanzees (Figure 6). Thus, with the exception of the specific changes described above, the CS in Laverania is highly conserved, including the observed motifs in the tandem repeat region, the latest contrast with other *Plasmodium* spp. found in mammals. [13,16,61,67].

#### Merozoite surface protein 2

**Figure 7:**
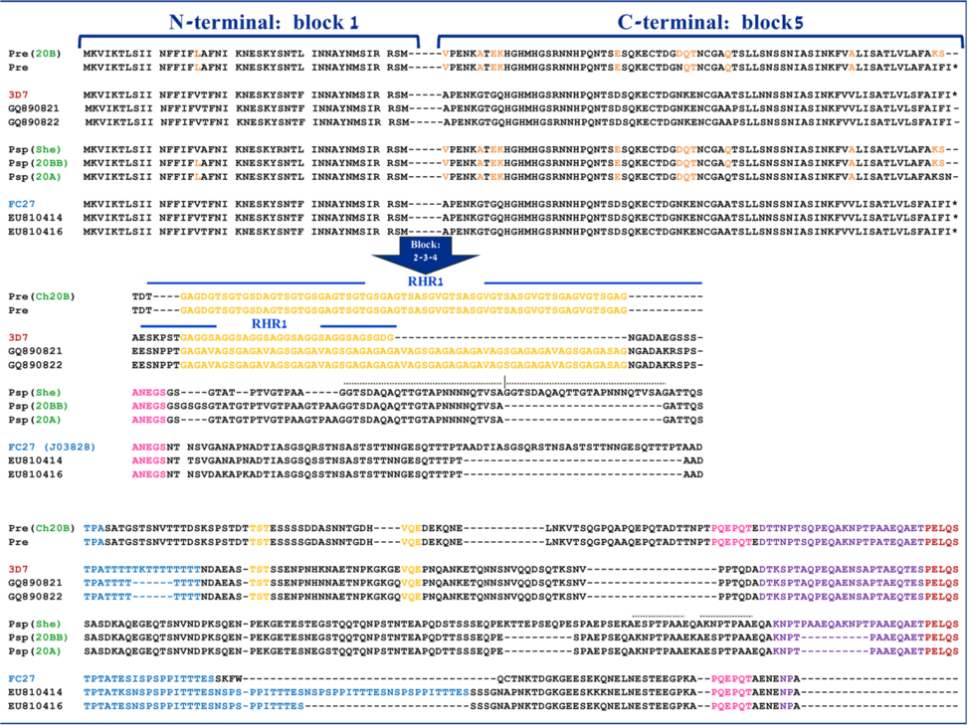
**Alignment of the N and C-terminal region for orthologous genes that encode MSP2 in the Laverania species.** The repeat homology regions 1 or RHR1 described by Rich and Ayala 2000 [44] are shown in yellow.

The two *msp2* allele families in *P. falciparum* are almost identical at nucleotide sites encoding the N and C termini [42], but they differ in a central low complexity region that clearly separates the two allele families into the so-called 3D7 and FC27 types, named after the strains from which they were originally sequenced [69]. It has been proposed that these two allele families originated after the *P. reichenowi*-*P. falciparum* split [44,70]. One line of evidence for this came from the so-called repeat homology regions or RHRs [44,70], where repeats observed in *P. reichenowi* showed similarities with one allele family or the other. Figure 7 depicts the amino acid alignment of the *msp2* allele families and the new sequences of *P. reichenowi* (isolate 20, mixed infection) and *Plasmodium* sp*.* (isolates Shegue and 20) parasites; these four were the only complete sequences obtained in this investigation. *Plasmodium reichenowi* shares with the 3D7 the so-called RHR1 marked in yellow [44] but it was not found in the *Plasmodium* sp. parasite or in the FC27 family. Attempts were made to identify the second RHR [44,70] (in blue) that is shared among *P. reichenowi* and *P. falciparum.* However, the patterns do not seem clear and whether it is actually homologous was difficult to determine. A conserved motif (in pink) is shared between FC27 alleles and *P. reichenowi.*

**Figure 8:**
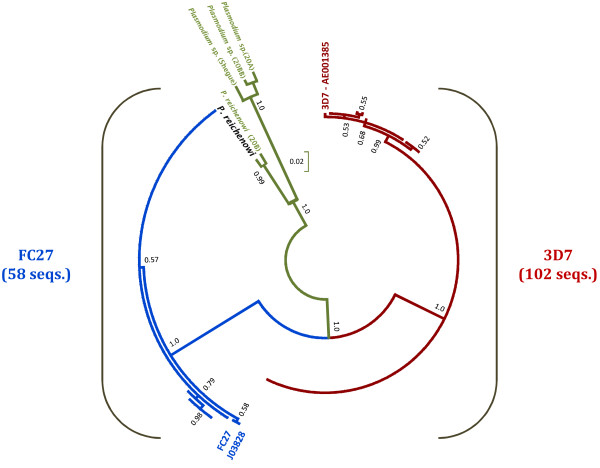
**Bayesian phylogenetic tree of the Laverania *Plasmodium *species based on the *msp2 *gene.** The values above branches are posterior probabilities (see methods). The two groups that are called allele families, 3D7 (in red) and FC27 (in blue) are shown.

The second line of evidence comes from the conserved flanking regions (Block 1 and 5 *sensu*[42]) where *P. reichenowi* seems separated from the two-allele families. However, no formal phylogenetic analyses were performed. Here a phylogeny of the newly sequenced *msp2* from chimpanzee parasites together with all complete *P. falciparum msp2* sequences was inferred (Figure 8). The chimpanzee lineages are different from the two-allele families. Indeed the *P. reichenowi* and the *Plasmodium* sp. alleles form a monophyletic group clearly different from the two *P. falciparum* allele families. Given the host switches observed, the time of origin for these two families cannot be estimated by assuming that *P. reichenowi* and *P. falciparum* diverged with their host as previously done [42].

#### Evolution of the var2CSA DBL1 domain

**Figure 9:**
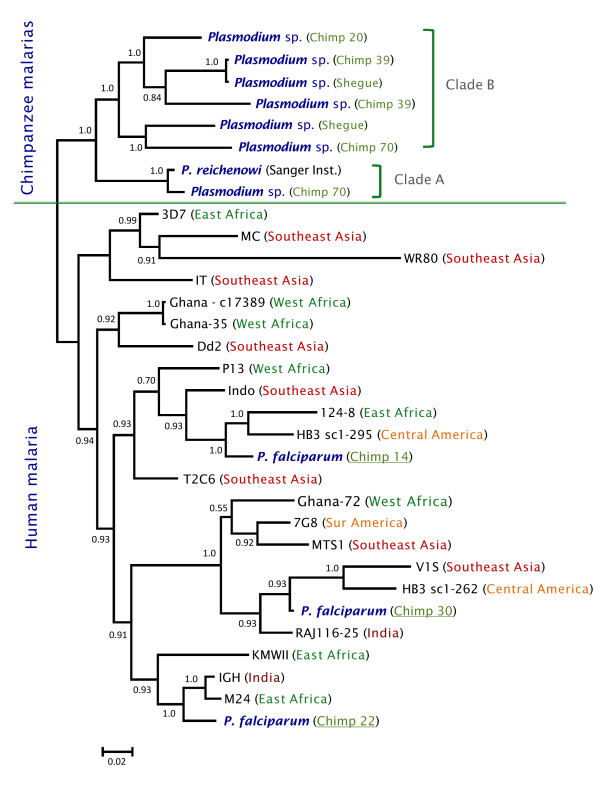
**Bayesian phylogenetic tree of the Laverania *Plasmodium *species based on the *var2CSA *DBL1 gene.** The values above branches are posterior probabilities (see methods).

The *var*2CSA DBL1 domain was not successfully amplified from the all the chimpanzee isolates; however, data was obtained from *P. billbrayi*, *P. gaboni,* the *Plasmodium* sp. lineage, *P. reichenowi* and three of the *P. falciparum* parasites. A Bayesian phylogeny is depicted in Figure 9; it includes all the sequences obtained in this investigation together with those available at the GenBank. As expected, the *P. falciparum* parasites found in chimpanzees clearly group within the known sequences from human infections. Although there are different malarial parasites present in chimpanzees, the *var*2CSA sequences did not show much divergence among them. In some cases, there is an apparent gene duplication as evidenced by the two sequences obtained from some of the isolates, e.g. the isolates that yield *P. gaboni* mtDNA (isolate 39) and *Plasmodium* sp. (isolate 70) (Figure 9). Nevertheless, given that these are field isolates, there is still the possibility of mixed infections even when those were no detected using other genes.

**Figure 10:**
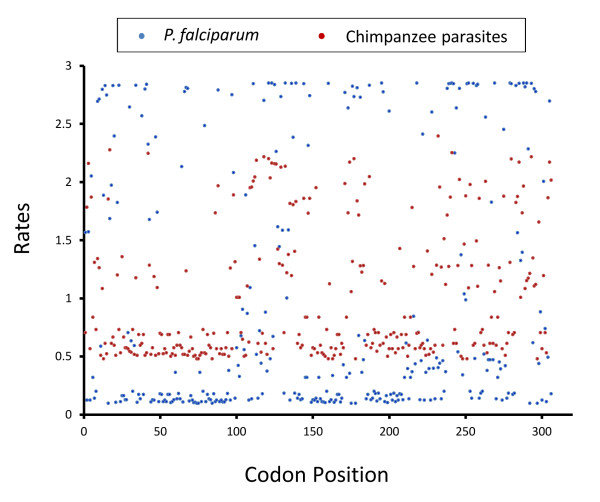
**Mean (relative) evolutionary rates for the VAR2CSA protein.** These rates are scaled such that the average evolutionary rate across all sites is 1. This means that sites showing a rate < 1 are evolving slower than average and those with a rate > 1 are evolving faster than average. These relative rates were estimated under the Jones-Taylor-Thornton model (+G). A discrete Gamma (+G) distribution was used to model evolutionary rate differences among sites (4 categories). Blue dots correspond with *P. falciparum* and red dots are chimpanzee parasites. The graph shows that *P. falciparum* sequences are by far more divergent that those lineages found in chimpanzees.

Table 4 shows estimates of the genetic diversity for the alleles reported here. The diversity of the DBL1 domain from *var*2CSA DBL1 in chimpanzees, even when all the sequences are considered as one “species”, was lower than the diversity found in *P. falciparum* (0.0939 ± 0.0062 vs. 0.1173 ± 0.0064 respectively). It is also worth noting that in almost all comparisons there were significantly more synonymous than non-synonymous substitutions (Table 4) suggesting functional constraint. The diversity within *P. falciparum* and those lineages found in chimpanzees was further explored using codon-based analyses. Figure 10 shows the evolutionary rate per codon. The estimate of evolutionary rates per codon shows that many *P. falciparum* codons (in blue) vary more than in the chimpanzee lineages (in red) (Figure 10). Overall, the results indicate that there is extensive variation within *P. falciparum* that is not observed among distinct ape malaria lineages. Thus, like in *msp2*, such divergence seems to be originated after the *P. falciparum-P. reichenowi* split.

**Table 4:** **Polymorphism found in *var2CSA *DBL1 gene sequences from *P. falciparum *and chimpanzee malarial parasites**

	**VAR2CSA-DBL1**				
**Species**	**π (SD.)**	**Ds**	**Dn**	**Ds-Dn (SD.)**	***p *(Z-stat)**
*P. falciparum* (Human, N = 21)	0.1173 (0.0064)	0.1674	0.1200	0.0471 (0.0186)	0.0227 (−2.3078) **Ds** > Dn
*P. falciparum* (Human-Chimp, N = 24)	0.1146 (0.0066)	0.1641	0.1171	0.0470 (0.0209)	0.0207 (−2.3449) **Ds** > Dn
Chimpanzee malaria (all Chimp, N = 8)	0.0939 (0.0062)	0.1424	0.0914	0.0509 (0.0206)	0.0087 (−2.6660) **Ds** > Dn
Chimpanzee malaria (Chimp-Clade A, N = 2)	0.0122 (0.0035)	0.0360	0.0069	0.0291 (0.0145)	0.0464 (−2.0124) **Ds** > Dn
Chimpanzee malaria (Chimp-Clade B, N = 6)	0.0895 (0.0065)	0.1240	0.0893	0.0347 (0.0188)	0.0797 (−1.7670) **Ds** = Dn

Clade A and B are shown in Figure 9.

## Discussion

### Defining *Plasmodium* species

The molecular evidence indicates that there are several species within the Laverania clade. These lineages have been independently reported [9-11,21] indicating that they are actively transmitted. Regardless of the absence of morphological evidence, these are well-defined phylogenetic species since they show a level of divergence comparable with those observed between related taxa such as *P. vivax* and *Plamodium cynomolgi*. The use of molecular methods for defining species in malarial parasites is not free of controversy [71], however, such debates are not new in biology [72].

There are several considerations favouring the use of molecular criteria to delimit species in *Plasmodium*. First, several infections appear to be sub-microscopic making the use of morphological evidence difficult if not impossible, in many cases. Second, in the case of Laverania parasites, whenever blood stages have been observed, they seem to be indistinguishable from *P. falciparum*[3,21,73]. Such limitations on the use of morphological traits are relatively common in malarial parasites. For example, trained microscopists could not detect *P. knowlesi* when observed in humans and misidentified these zoonotic infections as *P. malariae* or *P. falciparum*[14,74]. Furthermore, there is clear genetic divergence in the mtDNA among parasites morphologically identified as *Plamodium relictum* in birds [75,76]. Finally, there are many non-human primates that are endangered species, so studies on parasite biodiversity likely will use opportunistic samples that may only allow for DNA-based approaches [9-11,21,22]. It is worth noting that unlike malarial parasites in Asian apes or lemurs [3,13,25,52,73], there were not multiple *P. falciparum*-like morphologically described species in African apes. The only recognized species was *P. reichenowi*[3,52,73], a parasite from which there is molecular data, and these lineages are related, but clearly not the same parasite [9-11,21,22]. Thus, it is not necessary to establish whether these new species correspond with others previously described. Overall, the DNA evidence allows a stable taxonomy that facilitates comparisons among studies.

Separate discussions are what is considered a level of divergence that could indicate different species or subspecies [22,25,53,76,77] and what kind of genetic data should be used. For example, the use of 400-600 bp segments of the mitochondrial gene *cytb*, as has been widely used in avian malaria studies, seems insufficient. However, a suitable fragment of the mtDNA that includes the *cytb* and *cox1*[10] provides valuable phylogenetic signal that is consistent with the complete mtDNA and other genes [5,10,13,25]. In the context of this investigation, *P. gaboni* and *P. billbrayi* are a good case study [22]. The available evidence, at least at the level of complete mtDNA sequences, indicates that the divergence between *P. gaboni* and *P. billrayi* is comparable to that between *P. vivax* and *P. cynomolgi*. Accordingly, if *P. billbrayi* were considered a synonymy of *P. gaboni*, and consequently studied as part of a single taxon, then the observed mtDNA “polymorphism” would exceed that found within any other known *Plasmodium* species with worldwide distributions (Table 1). Such pattern will require an *ad hoc* explanation that cannot be supported on what is known about the mitochondrial genome diversity in *Plasmodium*. Thus, it seems more parsimonious to hypothesize the existence of two different species, rather than explaining how such a high level of polymorphism can be harboured within one species. The *P. gaboni*-*P. billbrayi* separation is supported by the *dhfr* and *csp* genes but some of those differences are hard to interpret in the absence of mtDNA data (e.g. differences in low complexity regions in the *csp*, see Figure 5). However, taking together the observed differences in the *dhfr* and *csp* genes and the mtDNA divergence, a recent but clear divergence between *P. gaboni* and *P. billbrayi* was found. Thus, the hypothesis that these two evolutionary lineages are different species seems to hold.

Likewise, the so-called *Plasmodium* sp. appears to be a distinct lineage that is closely related to *P. reichenowi;* whether these are species or sub-species require some additional considerations. The *Plasmodium* sp. lineage corresponds with mitochondrial haplotypes identified as *P. reichenowi* that were previously isolated from chimpanzees in the same region and reported as part of clade C1 [10], as well as with a previous study using complete mtDNA [9]. The differences in terms of the *csp* and the *var*2CSA are mostly qualitative and difficult to interpret. The *dhfr* as nuclear gene, however, separates *P. reichenowi* from *Plasmodium* sp. by only 11 SNPs (d = 0.007 versus d = 0.016 between *P. reichenowi* and *P. falciparum* using Kimura 2-parameter). This difference is hard to interpret since this gene is seldom sequenced in non-human primates so we lack a good database for comparison. Although the *msp2* actually separates these two lineages (*P. reichenowi* from *Plasmodium* sp.) in terms of its low complexity regions and SNPs in the 3′ and 5′ termini, it is an antigen under balancing selection [42]. Nevertheless, the differences on *dhfr* and *msp2* establish a linkage between nuclear and mitochondrial genes [77] suggesting that these two lineages are at least different sub-species. The divergence of these two *P. reichenowi s.l.* lineages seems to be the result, at least in part, of the geographic differentiation of the host populations (*P. troglodytes*) [10]. However, in order to set a high standard, additional data is needed in order to determine whether these should be considered sub-species or species. The reference mitochondrial haplotypes for this lineage called as *Plasmodium* sp. in this investigation are identified with the accession numbers GQ355476 and KF040083. At this point, it is worth noting that identifying species or sub-species is not only of taxonomic interest. Indeed, using “pooled data” from otherwise different species could affect comparative analyses aiming to ascertain the processes shaping the genetic diversity of *P. falciparum*. Furthermore, having delimited molecular lineages is epidemiologically critical in determining whether or not apes are a reservoir for *P. falciparum*[18].

### Apes as potential reservoirs for human malarias

The finding of chloroquine resistant *P. falciparum* in chimpanzees provides evidence of a host switch from humans at a time scale that is epidemiologically relevant. However, whether this indicates that chimpanzees could act as malaria reservoirs is a different matter. Indeed, there are several criteria that need to be fulfilled, such as evidence that apes can sustain *P. falciparum* infections and produce gametocytes [73,78], as well as understanding the ecological factors that may allow vectors to feed on apes and humans. It is worth noting that *P. falciparum* does not yield high parasitemias at least in chimpanzees [73,78], so it may be possible that the same phenomenon happens in gorillas. Thus, there is no evidence that currently confirms or categorically rules out apes as reservoirs for *P. falciparum*.

### Evolution of gene encoding malarial antigens

This investigation shows how ape malarias provide new insights on the evolution of important antigens. In the case of the CS protein, unlike other *Plasmodium* from mammals [13,16,59,61], the central tandem repeat region is conserved among the Laverania lineages. Whether this pattern is the result of immune pressures or functional constrains [62] from genomes with a trend toward accumulating asparagine rich low complexity regions [79], are issues that will require additional studies. Another observation is the conservation of peptides binding heparin sulphate (HS). Specifically, the two cell surface receptors involved in the sporozoite attachment to the hepatocytes (highly sulphated HS associated with region I + or **Pf2** and **Pf4** region II +, see Figure 1[63,64]) are almost identical among the Laverania lineages. These similarities suggest that the Laverania sporozoites can bind comparable receptors in the human and apes livers [59] providing additional support to the observation that these parasites can potentially infect several species of hominids.

Although Laverania parasites are the scope of this study, the lack of region I in *Plamodium gonderi* and the *Plasmodium* spp. should be highlighted. These are the only known *Plasmodium* species with a CS protein missing this functionally important region [16,38,63]. In the absence of additional information, any interpretation is speculative. However, this CS protein “natural knockout” of the region I (see [38,66]) suggests that these parasites may have some unique processing of their CS proteins and may provide new insights on the *Plasmodium* invasion of the liver.

Regarding the *msp2*, it has been proposed that these two major groups of alleles (also referred as allele families) originated soon after the *P. reichenowi*-*P. falciparum* split [42,44,70]. This contrasts with the allele families in *msp1* that are considered an old polymorphism [43]. The data presented here indicates that the diversity of *msp2* alleles observed in *P. falciparum* originated after the *P. falciparum*-*P. reichenowi* split. This hypothesis was originally proposed by the discovery of the so-called mosaic structure of *P. reichenowi* MSP2 [44,70], however, it was still possible that additional data from other Laverania species could provide evidence of an older polymorphism in *msp2*. Indeed, some motifs are conserved among the different allele families, and *P. reichenowi* and *Plasmodium* sp. parasites (Figure 7), suggesting that such convergence/recombination events predate the *P. reichenowi*-*P. falciparum* divergence. Here, a phylogeny (Figure 8) shows that the two-allele families are part of a monophyletic group separated from the chimpanzee parasites. Considering that *msp2* is only found in the Laverania clade, the fast divergence of these two alleles families may had been driven by allele-specific immunity [41,80] that could lead to a pattern consistent with balancing selection [42]. Recent high genetic diversity within extant *P. falciparum* populations seems to be also the case for *var*2CSA.

As described earlier, *var*2CSA is critical in pregnancy-associated malaria and is one of the few members of the *var* gene family that is found in all *P. falciparum* isolates [45-47]. This preliminary study shows that *var*2CSA can be found in relatively divergent Laverania lineages (Figure 9). Considering the fact that other *var* genes are seldom shared among even *P. falciparum* isolates [48,49], this observation reinforces the importance that this particular *var* gene has in *P. falciparum* and the other species of the Laverania clade. The second observation that emerges is that *var*2CSA accumulated extraordinary genetic polymorphism after the *P. reichenowi* and *P. falciparum* split. Whereas these observations should be considered preliminary, lineages that are highly divergent in the mtDNA (e.g. *P. gaboni*) show relatively low divergence in the DBL1 *var*2CSA*.* The results presented here also indicated that it is possible that *var*2CSA is duplicated in some chimpanzee lineages, a phenomenon that has been observed in some *P. falciparum* strains (e.g. HB3, see Figure 9). Taken together, this study shows that the presence of *var*2CSA is relatively “ancient” (e.g. shared between ape lineages and humans), a situation contrasting that seen with other *var* genes [49]. This indicates some functional importance for this *var*2CSA gene. Such evidence allows hypothesizing that natural selection has maintained this *var* gene in the Laverania group; thus, it should confer some adaptive advantage for the parasite*.* If acquired immunity that results from malaria exposure affects transmission in humans (parasite fitness), then *var*2CSA may increase the parasite transmissibility in a second segment of the population that otherwise will be immune, such as primigravid women. Unfortunately, there is no information on gametocytaemia in primigravid women or whether parasite transmissibility increases for that malaria high risk group. Thus, in the absence of such data, this hypothesis of a selective advantage for *var*2CSA cannot be formally tested.

## Conclusions

The Laverania clade consists of a highly diverse group of species. All the species described are actively transmitted; evidence of this is that they have been independently found in different studies. Thus, they are stable evolutionary and ecological units as expected for any *Plasmodium* species. This investigation provides additional evidence that chimpanzees can acquire *P. falciparum* from humans. However, whether they can sustain transmission and act as a reservoir is a matter that requires additional studies.

This study emphasizes the value of comparative studies on genes involved in the infection of humans by malarial parasites. Such studies improve our understanding of the molecular bases of these parasites pathogenesis and provide information on the rate and mode of evolution of antigens that are considered vaccine targets. As specific examples, this investigation provides evidence of constraints in the evolution of the CS protein among the Laverania species that have not been observed in other *Plasmodium* lineages*;* specifically, the conservation of its tandem repeat region. In contrast, antigens such as MSP2 and VAR2CSA likely accumulated genetic variation very recently, suggesting the role of natural selection and highlighting their importance as adaptations to the human host.

Studying the evolution of the *P. falciparum* clade will require an extensive sampling of parasites from African apes; sampling that is difficult due to ethical concerns and regulations limiting research on apes. In addition, collecting viable isolates of these *falciparum*-like parasites in order to use them for comparative studies may prove to be even more complex. Nevertheless, it is worthwhile to consider such studies if they will not expose African apes to any significant risk.

## Abbreviations

CIDRpam: A cysteine-rich inter-domain region; cox1: cytochrome oxidase subunit 1 gene; cox3: cytochrome oxidase subunit 3 gene; CSA: Chondroitin sulphate A binding in the placenta; CSP: Circumsporozoite protein; cytb: Cytochrome b gene; DBL: Duffy binding-like domains; dhfr-ts: Gene encoding dihydrofolate reductase-thymidylate synthase; GTR: General time reversible + gamma (G) or invariants (I); HKY + G: Hasegawa-Kishino-Yano + gamma model; HS: Heparan sulphate; ML: Maximum Likelihood; MCMC: Markov Chain Monte Carlo; Mya: Million years ago; MSP1: Merozoite Surface Proteins 1, MSP2, Merozoite surface proteins 2; mtDNA: Mitochondrial genomes; PAM: Pregnancy-associated malaria; PCR: Nested polymerase chain reaction; PfCRT: Chloroquine resistance transporter; PSRF: Potential scale reduction factor.

## Competing interests

The authors declare that they have no competing interests.

## Authors’ contributions

MP conducted the molecular genetic studies and analyzed the data. MC and KC provided the samples and contributed to the editing of the manuscript. AE supervised and directed the research and contributed to the writing and editing of the manuscript. All authors contributed in the project design. All authors read and approved the final manuscript.

## Supplementary Material

Additional file 1 Methods: Samples, PCR amplification, and time estimation. Click here for file

Additional file 2 List of primers used to amplify the different genes included in this study. Click here for file

Additional file 3 *Plasmodium* species included in our phylogenetic analysis. Click here for file

Additional file 4 A complete list of *Plasmodium* species included in all the analyses (*dhfr*, *csp*, *msp2* and *var*2CSA). Click here for file

Additional file 5 Beast node numbers for the *Plasmodium* phylogeny as used in Table 2. All calibration points used are shown. Click here for file
