# Supplementary material for: Malarial parasite diversity in chimpanzees: the value of comparative approaches to ascertain the evolution of Plasmodium falciparum antigens
Source: Malar J. 2013 Sep 17;12:328. doi: 10.1186/1475-2875-12-328 (PMC3848613; doi:10.1186/1475-2875-12-328)
Supplement: Additional file 1 — Methods: Samples, PCR amplification, and time estimation. [file 1475-2875-12-328-S1.docx]

**Additional file 1: Methods: Samples, PCR amplification, and time estimation.**

**Samples and diagnostic**

The chimpanzees are housed at the Jane Goodall Institute’s Tchimpounga Chimpanzee Rehabilitation Center (JGI) in Congo ([www.janegoodall.org](http://www.janegoodall.org)). Blood samples were collected throughout 2009-2010 by the veterinary staff as part of the chimpanzees’ routine health examinations following standards approved by the Pan Africa Sanctuary Alliance ([www.pasaprimates.org](http://www.pasaprimates.org)). The archived remainers of those blood draws were made available to Arizona State University (ASU) for malaria diagnostics by the JGI. The samples were exported by the JGI under the Congo CITES Export Permit No. 001 (Feb 2, 2010) and imported by ASU under U.S. CITES Import Permit No. 09US094332/9. Other samples used in this investigation were described elsewhere [9].

Genomic DNA was extracted from the remainders (approximately 200 µl of whole blood) using the QIAamp® DNA Blood Mini kit (Qiagen, GmbH, Hilden, Germany) and each sample was screened for *Plasmodium* parasites by nested polymerase chain reaction (PCR), using primers for a 1,200 bp fragment of the Cytochrome b (cytb) gene. The cytb external primers were Forward 5’ TGT AAT GCC TAG ACG TAT TCC 3’/Reverse 5’ GT CAA WCA AAC ATG AAT ATA GAC 3’ and the internal primers were Forward 5’ T CTA TTA ATT TAG YWA AAG CAC 3’/Reverse 5’ G CTT GGG AGC TGT AAT CAT AAT 3’. The primary PCR amplifications were carried out in a 50 µl volume reaction using 20 ng of total genomic DNA, 3 mM MgCl_2_, 1X PCR buffer, 1.25 mM of each deoxynucleoside triphosphate, 0.4 mM of each primer, and 0.03 U/µl AmpliTaq polymerase (Applied Biosystems, Roche-USA).

The nested PCRs were also made in a 50 µl volume reaction using only 1 µl of the primary PCRs, 1.5 mM MgCl_2_, 1X PCR buffer, 1.25 mM of each deoxynucleoside triphosphate, 0.4 mM of each primer, and 0.03 U/µl AmpliTaq polymerase. The primary PCR conditions were: partial denaturation at 94ºC for 4 min and 36 cycles of 1 min at 94ºC, 1 min at 53ºC and 2 min extension at 72ºC, with a final extension step of 10 min at 72ºC added in the last cycle. The nested PCR conditions were: partial denaturation at 94ºC for 4 min and 30 cycles of 1 min at 94ºC, 1 min at 56ºC and 2 min extension at 72ºC, with a final extension step of 10 min at 72ºC added in the last cycle. Both strands for all the cytb fragments were sequenced directly, using an Applied Biosystems 3730 capillary sequencer, and identified as *Plasmodium* using BLAST showing significant similarity with different malaria parasites species found in apes.

**Species diversity and phylogenetic analyses**

Approximately 5,800 bp of the parasites’ mitochondrial genomes (mtDNA) and the gene encoding dihydrofolate reductase-thymidylate synthase (*dhfr-ts*) from all positive samples were amplified. In the case of mtDNA, we used the oligos Forward 5’ GA GGA TTC TCT CCA CAC TTC AAT TCG TAC TTC 3’/Reverse 5’ CAG GAA AAT WAT AGA CCG AAC CTT GGA CTC 3’ with TaKaRa LA TaqT^M^ Polymerase (TaKaRa Mirus Bio Inc). The PCR conditions were: partial denaturation at 94ºC for 1 min and 30 cycles of 30 sec at 94ºC, 7 min at 68ºC and a final extension step of 10 min at 72ºC. To detect mixed infections, in all cases, at least two independent PCR products were purified using QIAquick® Gel extraction kit (Qiagen, GmbH, Hilden, Germany), cloned in the pGEM®-T Easy Vector systems (Promega, USA), and a minimum of four clones and both strands were sequenced from each individual [13,25].

In order to contrast with a gene from the nucleus, the gene encoding dihydrofolate reductase-thymidylate synthase (*dhfr-ts*) was also obtained for different *Plasmodium* species by PCR using degenerate primers Forward 5’ G CMA TAT GTG CAT GTT GTA ARG 3’/Reverse 5’ GC CAT ATC CAT TKA AAT TTT WTC 3’. The PCR was carried out in a 50 µl volume reaction using 20 ng of total genomic DNA, 2.5 mM MgCl_2_, 1X PCR buffer, 1.25 mM of each deoxynucleoside triphosphate, 0.4 mM of each primer, and 0.03 U/µl AmpliTaq polymerase. The PCR conditions were: partial denaturation at 94ºC for 4 min and 36 cycles of 1 min at 94ºC, 1 min at 55ºC and 2 min extension at 72ºC, and a final extension step of 10 min at 72ºC added in the last cycle. For each sample, at least two independent PCR products were also purified, cloned, and a minimum of three clones were sequenced from each individual.

Independent alignments for nucleotide sequences of the mtDNA and *dhfr-ts* gene were made using ClustalX v2.0.12 and Muscle as implemented in SeaView v4.3.5 with manual editing. A list of the species included in phylogenetic analyses is provided in the Additional file 1.

In the *dhfr-ts* analysis, sequences from different *Plasmodium* species available in GenBank were included (see Additional file 4: AY033582, GQ369532-GQ369535, AL844503, EU046230, XM_740285, M30834, U12275, AY846633, EF188274, AM910987, DQ514920).

**Estimation of divergence times**

Time trees were estimated using BEAST v1.6 [29] on the nucleotide mtDNA sequences using four categories: each gene (*cox1, cox3, cytb*) plus the non-coding regions as a separate partition. Relaxed clock methods were applied with a GTR+G model of substitution with heterogeneity among sites and in the four partitions, optimizing the parameters specifically for each of the partitions. Uniform distributions were used as priors for the calibration intervals and the MCMC was carried out until convergence and good-mixing of the samples were reached [29]. Since time estimates are sensitive to the assumptions and methods utilized [13,25], the exploration of different scenarios is always recommended [25].

In this investigation three events were used as calibration points, one based on a biogeographical landmark and two from fossils. The biogeographic event is the colonization of Madagascar by terrestrial mammals. This process took place during a relatively short timeframe around ~65-20 Mya, in the Cenozoic [30,31]. The assumption is that malaria was introduced by a terrestrial mammal and, consequently, times younger than ~20 Mya for the origin of lemur malarias are unlikely. The second calibration assumes that African parasites found in *Mandrillu*s spp. and *Cercocebus* spp. [32-34] diverged from those *Plasmodium* spp. found in Southeast Asian macaques when *Macaca* branched from *Papio* [33,34]. Fossils identified as *Macaca* spp. indicate that such an event took place 6-8 Mya as minimum boundaries [32]. This fossil calibration is considered conservative [25]; thus a relaxed version that includes molecular estimates allowing for a broader interval of 6-14.2 Mya was also considered [25]. Finally, a third calibration point used was the human/Macaca split (23.5 Mya) [35] for the divergence of *P. malariae* from the lineage leading to the Southeast Asian malarias [25].

The scenarios explored, based on the calibration points described above, were: (1) a combination of the relaxed 6-14.2 Mya calibration for *Papio-Macaca* divergence with the minimum of 20 Mya for the origin of lemur parasites; (2) a combination of the 6-14.2 Mya calibration with the two the minimums: 23.5 Mya for the human/Macaca split and 20 Mya; and (3) the most conservative time period of 6-8 Mya narrowly defined around the fossils of the *Papio-Macaca* divergence with a minimum of 20 Mya for the origin of the lemur lineage.

***Plasmodium falciparum* chloroquine resistance transporter**

In order to ascertain whether the *P. falciparum* strains found in chimpanzees were acquired from humans, the chloroquine resistance transporter (*Pfcrt*) was sequenced so that mutations conferring resistance to the drug could be detected. A fragment of approximately 250 bp was amplified by PCR using degenerate primers Forward 5’ TTT TTC CCT TGT CGA CCT TAA C 3’/ Reverse 5’ GAA TAA AAC AAT AAA GAA CAT AAT CAT AC 3’. This fragment contained all the mutations that have been associated previously with chloroquine resistance (C72**S**, M74**I**, N75(**E/K**) AND K76**T**) [36]. The PCR was carried out in a 50 µl volume reaction using 20 ng of total genomic DNA, 2 mM MgCl_2_, 1X PCR buffer, 1.25 mM of each deoxynucleoside triphosphate, 0.4 mM of each primer, and 0.03 U/µl AmpliTaq polymerase. The PCR conditions were: partial denaturation at 94ºC for 4 min and 36 cycles of 1 min at 94ºC, 1 min at 56ºC and 2 min extension at 72ºC, and a final extension step of 10 min at 72ºC added in the last cycle. The PCR products were purified and sequenced directly from each individual. An alignment of nucleotide sequences of the *Pfcrt* gene was made using ClustalX v2.0.12 and Muscle as implemented in SeaView v4.3.5 with manual editing.

**Malaria antigens**

***Circumsporozoite protein* (CSP)**

CSP is the predominant protein found on the surface of the sporozoite, the haploid stage that is inoculated by the mosquito vector into the vertebrate host. It is a very complex protein with a central tandem repeat containing a immunodominant B cell epitope that acts as a “smoke screen” hampering the ability of the immune system to target more important segments of the protein that are also exposed [37]. CSP has fundamental roles in the development of the diploid stage in the mosquito vector (oocyst) and the first merozoites (red blood stage) in the liver prior to the start of the erythrocytic cycle [37,38]. In the liver, the tissue ligand for CSP is glycosaminoglycan heparin sulfate (HS) found on the surface of hepatocytes. All these characteristics make CSP a major target for pre-erythrocytic stage vaccines aiming to elicit sterile immunity [38,39]. In this investigation, the orthologous genes encoding the CSP protein in malarial parasites from chimpanzees were compared against those sequences available in the GenBank (See additional file 4: U65959, P13815, P06914, P06915, JQ308499, P02894, M15100, JQ308506, AFI80538, JQ308498)[13,16]. PCR was carried out in a 50 µl volume reaction using 20ng of total genomic DNA, 2.5 mM MgCl_2_, 1X PCR buffer, 1.25 mM of each deoxynucleoside triphosphate, 0.4 mM of each primer, and 0.03 U/µl AmpliTaq Gold® DNA polymerase (Applied Biosystems, Roche-USA). The PCR conditions were: partial denaturation at 94ºC for 4 min and 36 cycles of 1 min at 94ºC, 1 min at 56-58ºC and 2 min extension at 72ºC, and a final extension step of 10 min at 72ºC added in the last cycle. The primers used were: Forward 5’ A TGA TGA GAA AAT TAG CTA TTT TAT CTG 3’/Reverse 5’ C TAA ATA AGG AAC AAG AAG GAT AAT ACC 3’. In addition, CSP encoding genes from *P. fragile*, *P. gonderi* and *Plasmodium* sp. from mandrill were also amplified using similar protocols and degenerate oligos Forward 5’T ATA TAC MAG AAC AAG ATG AAG 3’/Reverse 5’ GG ATR TCA GCT ACT TAA TTG 3’. In all cases, at least two independent PCR products were purified and cloned; a minimum of three clones and both strands were sequenced from each individual.

The alignment of *csp* sequences was done using only the N and C-terminal regions since those can be accurately aligned, and the phylogenetic relationships were estimated following the methodology described for mtDNA with a general time reversible+invariant model (GTR+I). Bayesian support for the nodes was inferred in MrBayes using 2 x10^6^ Markov Chain Monte Carlo (MCMC) steps.

***Merozoite surface protein 2***

Merozoite surface protein 2 (MSP2) is a highly abundant GPI-anchored protein of *P. falciparum* that is found exclusively in the Leverania clade [9, 40-42]*.* This investigation compared the available *msp2* alleles in the GenBank from the two families against orthologs found in malarial parasites from chimpanzees (See additional file 4).

The orthologous gene encoding MSP2 was amplified by PCR using AmpliTaq Gold® DNA polymerase (Applied Biosystems, Roche-USA) and the following primers: Forward 5’ ATG AAG GTA ATT AAA ACA TTG TC 3’/Reverse 5’ TGG CAA AAG CTA AAA CAA GTG TTG C 3’. PCR conditions were: partial denaturation at 94ºC for 4 min and 36 cycles of 1 min at 94ºC, 1 min at 55ºC and 2 min extension at 72ºC, and a final extension step of 10 min at 72ºC added in the last cycle. For each individual, the amplicon was purified, cloned, and sequenced for a minimum of three clones from at least two independent PCR products. The alignment and phylogenetic analyses followed the same methodology as above; however, the data fitted a Hasegawa-Kishino-Yano+gamma model (HKY+G) using MEGA v5.0 [28]. Bayesian support for the nodes was inferred in MrBayes using 10 x10^6^ Markov Chain Monte Carlo (MCMC) steps.

***Var2CSA protein***

Pregnancy-associated malaria (PAM) is related with the expression of a *var* gene, known as *var*2CSA. Unlike others members of the *var* gene family, it is found in all *P. falciparum* parasites isolates [45,46]. VAR2CSA appears to be one of the few proteins mediating chondroitin sulfate A (CSA) binding in the placenta [45-47]; it is characterized by six Duffy binding-like (DBL) domains, a cysteine-rich inter-domain region (CIDRpam) module and short inter-domain regions [48,49]. In this investigation, approximately 1,500 bp (out of 10,000bp) of the *var*2CSA gene containing the complete Duffy binding-like 1 (DBL1) were amplified from seven malaria chimpanzee isolates. The PCR was carried out by using AmpliTaq Gold® DNA polymerase (Applied Biosystems, Roche-USA) and the following oligos: Forward 5’ ATA GAC AAA AGG TGT GGG AAG 3’/Reverse 5’ ACT AYC ATT AGA ACT AGA TCC 3’. PCR conditions were: partial denaturation at 94ºC for 4 min and 36 cycles of 1 min at 94ºC, 1 min at 50-55ºC and 2 min extension at 72ºC, and a final extension step of 10 min at 72ºC added in the last cycle. At least two independent PCR products were also purified, cloned, and a minimum of three clones were sequenced from each individual.

Alignment of DBL1 *var*2CSA nucleotide sequences, obtained from chimpanzees and those available in the GenBank (See additional file 4), was done using ClustalX v2.0.12 and Muscle as implemented in SeaView v4.3.5 with manual editing. The phylogenetic relationship among DBL1 *var*2CSA alleles was estimated as explained above with a general time reversible + gamma model (GTR+G). In this case, Bayesian support for the nodes was inferred using 20 x10^6^ MCMC steps.
