# Supplementary figures and images for: Malarial parasite diversity in chimpanzees: the value of comparative approaches to ascertain the evolution of Plasmodium falciparum antigens
Source: Malar J. 2013 Sep 17;12:328. doi: 10.1186/1475-2875-12-328 (PMC3848613; doi:10.1186/1475-2875-12-328)

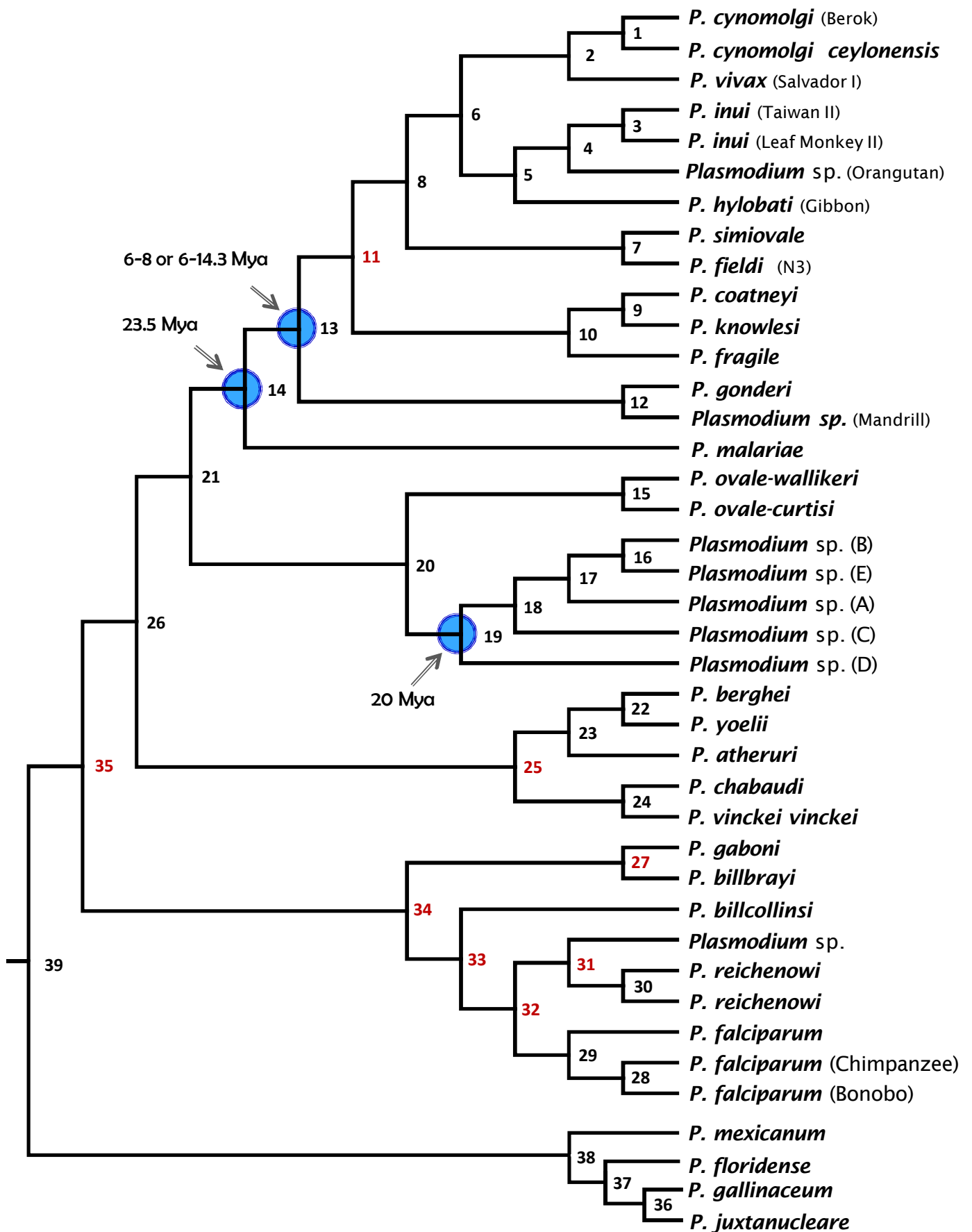

Additional file 5. BEAST node numbers for the *Plasmodium* phylogeny as used in Table 2.

Supplement: Additional file 5 — Beast node numbers for the Plasmodium phylogeny as used in Table 2. All calibration points used are shown. [file 1475-2875-12-328-S5.pdf]
